# Enhanced generation of iPSCs from older adult human cells by a synthetic five-factor self-replicative RNA

**DOI:** 10.1371/journal.pone.0182018

**Published:** 2017-07-27

**Authors:** Naohisa Yoshioka, Steven F. Dowdy

**Affiliations:** Department of Cellular & Molecular Medicine, UCSD School of Medicine, La Jolla, California, United States of America; University of Minnesota Medical Center, UNITED STATES

## Abstract

We previously devised a polycistronic, synthetic self-replicating RNA (srRNA) to generate human induced Pluripotent Stem Cells (iPSCs) that simultaneously expresses four reprogramming factors (4F). However, while the best 4F srRNA efficiently generated iPSCs from young fibroblasts, it was inefficient on adult human fibroblasts (>50 years). To increase the iPSC generation efficiency, we included additional reprogramming factors. We found that a single transfection of a five factor (5F) srRNA, containing *OCT4*, *KLF4*, *SOX2*, *GLIS1* and *c-MYC*, robustly generated iPSCs from adult human fibroblasts aged 54 to 77 and from a 24 year old cardiomyopathy patient donor. Interestingly, 5F-srRNA induced *LIN28A*, which was one of the original reprogramming factors. 5F-srRNA also accelerated the generation of iPSCs by seven days compared to 4F-srRNAs. Further improvements include phosphatase treatment to remove 5' phosphate and use of Lipofectamine MessengerMAX that increased transfection efficiency to ~90%. Together, these improvements enabled us to efficiently generate iPSCs from human fibroblasts using 5F-srRNA while eliminating both puromycin selection and feeder cells.

## Introduction

Induced pluripotent stem cells (iPSCs) were originally generated by infection of retroviruses expressing *OCT4*, *SOX2*, *KLF4* and *cMYC*, or *OCT4*, *SOX2*, *NANOG* and *LIN28* in mouse and/or human fibroblasts [1–3], which opened the door for regenerative medicine and cell-based therapies. However, iPSCs generated with the retroviruses have the potential for tumor development by insertional mutations of retrovirus genomes combined with the potential for latent reprogramming factor gene activation, especially *c-MYC* [4–7]. Therefore, for therapeutic purposes, integration-free methods, including Adenoviruses, episomal DNA plasmids, proteins, Sendai viruses, mRNAs, miRNAs, and self-replicating RNAs (srRNAs), were developed for generating human iPSCs. The advantages and disadvantages of which have been discussed in reviews [8–10]. Of these methods, RNA-based iPSCs approaches using Sendai viruses (a negative sense, single-stranded RNA virus), miRNAs, mRNAs and srRNAs avoid potential integration problems associated with DNA-based approaches and are inherently safer methods for future clinical applications.

We previously developed an srRNA system for generating human iPSCs by expressing the reprogramming factors using the RNA replicon of Venezuelan Equine Encephalitis (VEE) virus [11]. The VEE replicon is a positive sense, single-stranded RNA that mimics cellular mRNA with a 5’ cap and poly (A) tail [12]. We synthesized a srRNA containing non-structural proteins of VEE virus (RNA replicase) and iPSC reprogramming factors as a polycistronic synthetic srRNA *in vitro*. A single srRNA transfection resulted in extended expression of four reprogramming factors (4F-srRNA: *OCT4*, *KLF4*, *SOX2*, and *GLIS1* or *c-MYC* [OKS-iG or OKS-iM]) in the presence of B18R protein. Because exposure of cells to single-stranded RNA, including srRNAs, induces a strong IFN-α/β innate immune responses [11, 13, 14], culturing srRNA transfectants in the presence of B18R, a Western vaccinia virus protein that binds to and neutralizes type I interferons (IFNs), subdues the IFN response and allows for retention and replication of the srRNA.

Although 4F-srRNAs (OKS-iM and OKS-iG) worked well to generate iPSCs from both neonatal and adult human fibroblasts, the efficiency varied depending on the cell source, age of cells and/or four factor combinations. To address these problems, here we sought to increase the efficiency for iPSC generation by adding new factor(s) into the 4F-srRNA. We found that five factor srRNA (5F-srRNA), including *OCT4*, *KLF4*, *SOX2*, *GLIS1* and *c-MYC* (OKSi-GM), significantly increased iPSC generation in six different adult human fibroblasts, including old adult fibroblasts and a cardiomyopathy patient donor. Moreover, the time to reprogramming by 5F-srRNA was shortened by a week compared to 4F-srRNAs. Lastly, by improving srRNA quality with phosphatase treatment and using a new transfection reagent, Lipofectamine MessengerMAX, a single 5F-srRNA transfection resulted in ~90% efficiency of iPSC generation. Together, these improvements enabled the generation of iPSCs without the need for puromycin selection or feeder cells.

## Materials and methods

### Cell culture and media

BJ foreskin fibroblasts were obtained from ATCC and primary human foreskin fibroblasts (HFF) were kindly obtained from M. Haas (UCSD). FB#31 (male, age 55, healthy donor), FB#32 (female, age 54, healthy donor), and FB#33 (male, age 24, cardiomyopathy donor) fibroblasts were obtained from N.C. Chi (UCSD). HFB (female, age 77) human adult fibroblasts were obtained from L.S.B. Goldstein (UCSD). NHDFc (female, age 50) and HDF (male, age 56) human dermal fibroblasts were obtained from PromoCell (c-12302) and Cell Applications (106-05a), respectively. All fibroblasts were cultured in DMEM containing 10% FBS, MEM Non-Essential Amino Acids (NEAA), Pyruvate, penicillin, and streptomycin. HUES-9 and iPSCs were cultured with ES culture medium in Knockout D-MEM containing 20% Knockout SR, GlutaMAX, NEAA, 2-Mercaptoethanol, penicillin, streptomycin, and bFGF (10 ng/ml). Advanced DMEM containing 10% FBS, GlutaMAX, penicillin, and streptomycin was transiently used for iPSC reprogramming. STO feeder cells were prepared by mitomycin C treatment (10 μg/ml, Sigma). Matrigel (BD Bioscience) or Laminin-521 coated wells and conditioned medium of STO feeder cells were used for feeder free culture.

### Plasmids construction

T7-VEE-OKS-iM, T7-VEE-OKS-iG and (T7-VEE-Gfp) were previously constructed as described [11]. The *LIN28A* (BC028566) cDNA was obtained from ATCC. *LIN28A*, *cMYC* and *GLIS1* ORFs were cloned into T7-VEE-OKS by inserting the P2A sequence between them preceded by an IRES sequence yielding T7-VEE-OKS-iML, T7-VEE-OKS-iGL, T7-VEE-OKS-iGM and T7-VEE-OKS-iGML. PCR primers for cDNA amplification and P2A oligo sequences are listed in S1 Table.

### RNA synthesis

RNA synthesis was performed as described [11]. Briefly, VEE plasmids were linearized with MluI digest for the templates of RNA synthesis. The synthesis of srRNAs was performed with the RiboMAX Large Scale RNA Production System-T7 (Promega) kit for 2 hr incubated at 37°C. For 5’-Capping, srRNAs (70 μg) were incubated in ScriptCap Capping enzyme (CELLSCRIPT) and ScriptCap 2’-O-Methyltransferase (CELLSCRIPT) for 45 min at 37°C. After 5’-Capping, an additional ~150 bases of poly(A) tail was added by Poly(A) Polymerase (CELLSCRIPT) for 30 min at 37°C. srRNAs were then treated with the Antarctic phosphatase (NEB) for 30 min at 37°C. Following purification and precipitation with the 2.5 M ammonium acetate, srRNAs were resuspended in the RNA Storage Solution (Ambion) at 1 μg/μl concentration and stored at -80°C.

### iPSC generation with lipofectamine 2000 transfection

Cells were passaged on 6-well plate on day 0 and cultured to 80–100% confluency on day 1. To minimize the interferon response, cells were pretreated with 20% B18R conditioned media (BR-CM) for 20 min before transfection (100% B18R-CM containing 10% FBS was diluted with DMEM, and obtained 20% B18R-CM containing 2% FBS). Cells were transfected with 1 μg of each srRNA or co-transfected with srRNA and B18R mRNA (1:1 ratio, 1 μg RNA/well) in the presence of 20% B18R-CM. After 3 hr, transfection medium was changed to the Advanced DMEM containing 20% B18R-CM. On day 7, Advanced DMEM was replaced to ES culture medium containing 20% B18R-CM. Where used, puromycin (0.8 μg/ml) was added from day 2 to 10. Cells were passaged onto STO feeder cells with several dilutions on day 10 and cultured in ES culture medium containing 20% B18R-CM. Culture media containing 20% B18R-CM were changed every day until iPS cell colonies were generated. For generating iPSC in feeder free condition, cells on day 10 were passaged to Laminin-521 (LN-521, BioLamina) or Matrigel (#354277, Corning) coated 6-well plate with several dilutions in STO feeder conditioned medium containing 20% B18R-CM and bFGF (10 ng/ml). STO feeder conditioned medium was prepared from one day culture of STO feeder cells in ES culture medium (without bFGF and B18R-CM), filtrated and store at -20°C until use. For puromycin-free selection, cells were passaged in STO feeder with 6 times dilutions in the Advanced DMEM containing 20% B18R-CM on day 5. ES culture medium was used from day 7. Rock inhibitor (Y-27632, 5 μM) was added into ES culture medium when cells were passaged in STO feeder or feeder free conditions.

### iPSC generation with lipofectamine messengerMax (mMax) transfection

The maximum transfection efficiency with Lipofectamine MessengerMax (mMax; Thermo Scientific) was obtained with serum free condition (data not shown). Cells were passaged on 6-well plate on day 0 and cultured to 50 or 100% confluency on day 1. Cells were washed once with DMEM (no antibiotics and no serum) and then added DMEM (1 ml/well, 6-well) prior to transfection. For serum-free srRNA transfection, we used B18R mRNA to suppress the IFN responses and omitted the 20% B18R-CM treatment. Transfection complex with 5F-srRNA (1 μg), B18R mRNA (1 μg) and mMax reagent (6 μl) for one well of 6-well were prepared according to the manufacture’s protocol except for omitting the 10 min incubation of diluted messengerMax reagent and transfected into cells in the absence of serum. No incubation of the diluted messengerMax is significantly affect on the transfection efficiency of srRNAs. After 3 hr incubation, medium was changed to the Advanced DMEM containing 20% B18R-CM. 4 hr incubation will increase transfection efficiency for mMax reagent in current protocol (data not shown). For puromycin-free condition, cells were passaged to Matrigel or STO feeder on day 3 or day 5 with 6 or 12 times dilutions. ES culture medium was used from day 5.

### Immunoblotting

Cells were lysed with 2x RIPA buffer containing 0.3 M NaCl, 80 mM Tris-HCl (pH 7.5), 0.4% SDS, 2% Triton-X 100, 2% sodium deoxycholate, 100 μg/ml phenylmethylsulfonyl fluoride (PMSF), aprotinin (5 μg/ml) and leupeptin (5 μg/ml). Equal amount of proteins (~20 μg) were used for 9% SDS-PAGE and electroblotted onto a Nitrocellulose membrane. Membranes were incubated with primary antibodies O/N at 4°C after blocking with 4% Milk in PBS-T (0.05% Tween-20), and incubated with horseradish peroxidase-conjugated anti-rabbit, goat or mouse IgG (Santa Cruz). Protein bands were visualized using the ECL reagent (SuperSignal West Pico, Thermo Scientific).

### Antibodies

Antibodies used in this research are as follows: anti-OCT4 (sc-9081), anti-KLF4 (sc-20691), anti-GLIS1 (sc-67584), anti-c-MYC (sc-42), TRA-1-60 (sc-21705), SSEA1 (sc-21702), SSEA4 (sc-21704), anti-mouse (sc-2005), anti-rabbit (sc-2004) and anti-goat (sc-2020) from Santa Cruz; anti-SOX2 (AF2018) and anti-NANOG (AF1997) from R&D Systems; anti-LIN28A (#46020) from Abcam; anti-TRA-1-81 (09–0011) from Stemgent; Alexa Fluor 488 anti-mouse (A11029), Alexa Fluor 488 anti-rabbit (A11034) and Alexa Fluor 488 anti-goat (A11055) for immunostaining of iPS clones from Life Technologies. IRDye800 anti-mouse (610-132-121) for Odyssey imaging of iPSC colonies from Rockland.

### Primary iPSC colony staining with alkaline phosphatase, TRA-1-60, TRA-1-80, SSEA4 or SSEA1

After the generation of primary iPSC colonies, cell were washed with AP buffer (100 mM Tris, 100 mM NaCl, 50 mM MgCl_2_, pH 9.5) and stained with AP-Staining solution (1 mg/ml of Fast Red TR Salt hemi salt, 0.4 mg/ml of 1-Naphthyl phosphate disodium salt) in AP buffer for 15 min. For TRA-1-60, TRA-1-80, SSEA4 or SSEA1 staining, AP stained cells were then fixed in 4% paraformaldehyde for 10 min, and then treated with 0.1% Triton X-100 for 10 min. Cells were blocked with 2% BSA for 1 hr, and then incubated with primary antibodies in PBS at 4°C overnight. Cells were washed and incubated with IRDye 800 (1:800 dilutions) for 3 hr. Colonies were scanned with Odyssey imaging system (LI-COR).

### Immunofluorescence staining

Cells were washed twice with PBS and fixed in 4% paraformaldehyde for 10 min. Washed cells were treated with 0.1% Triton X-100 in PBS for 10 min. Cells were blocked with 2% BSA for 1 hr at room temperature (RT), and then incubated with primary antibodies in PBS at 4°C overnight. Cells were washed and incubated with secondary antibodies followed by incubation with Hoechst 33342, and then washed and stored in PBS. Primary antibodies such as rabbit anti-OCT4, goat anti-NANOG and anti-SOX2, mouse anti-SSEA4, anti-TRA-1-60 and anti-TRA-1-81 antibodies were used at 1:100 to 1:500 dilutions. Alexa Fluor 488 (BD Biosciences) secondary antibodies were used at 1:800 dilutions.

### Detection of srRNA in iPSC clones

Total RNAs were isolated with TRIzol (Life Technologies). For RT-PCR detection of srRNA, cDNAs were synthesized with iScript cDNA synsethis kit (Bio-Rad) from 1 μg of total RNA. 2 μl of 20 μl RT reaction was used for PCR amplification. PCR was performed with Taq DNA plolymerase (NEB) supplemented with PCRx enhancer (Life Technologies): 3 min at 94°C for initial denature; 36 cycles of 94°C for 25 sec, 56°C for 25 sec, 68°C for 30 sec; followed by 72°C for 5 min. Primers used for RT-PCR are described before [11]. For TaqMan RT-PCR detection, 10 ng of total RNA was used for TaqMan RT-PCR reactions. The nsP1 probe for TaqMan PCR was kindly gifted from Life Technologies. Standard curve was generated with 0.1 fg to 1 ng of 5F-srRNA (Slope: -3.60, R^2^ = 0.999), and then amount of 5F-srRNA in total RNA was calculated.

### qRT-PCR for ES markers

Total RNAs of iPSCs clones, HUES-9 and BJ were isolated with TRIzol (Life Technologies). TaqMan RT-PCR reactions were carried out using RNA-to-Ct one-step reaction (Applied Biosystem) according to manufacturer’s protocol. 10 ng of total RNA were used per reaction. Primers and probes were obtained from AB TaqMan Gene Expression Assay catalog (GAPDH, Hs99999905_m1; POU5F1 Hs03005111_g1; SOX2 Hs01053049_s1; DNMT3B Hs00171876_m1; TERT Hs00972656_m1; LIN28A Hs00702808_s1; NANOG Hs02387400_g1; TDGF1 Hs02339499_g1). Quantitative PCR reactions were carried out in triplicate, and conditions were as followed: 20 min 55°C, 10 min 95°C, 40 cycles of 95°C for 0.15 min, 65°C for 1 min. Data were analyzed on the 7300 real-time PCR system (Applied Biosystems) using the delta-delta Ct method.

### Teratoma formation

iPSC clones were intramuscularly or subcutaneously injected into the hind limb muscles or dorsal flank of NRG mice (approximately 10 cm dish cultured cells for 1 injection). After 2 to 3 months of injection, tumors were dissected and fixed with 4% paraformaldehyde, embedded into paraffin, and sectioned for hematoxilin and eosin (H&E) staining.

### Experimental oversight

All animal experiments were performed with the approval of the Institutional Animal Care and Use Committee of University of California, San Diego (protocol number S01103). Studies using human ESCs and hiPSCs were approved by the IRB/ESCRO committee at University of California, San Diego (project number 071552ZF).

## Results

### Construction of 5F-srRNA

**Fig 1:**
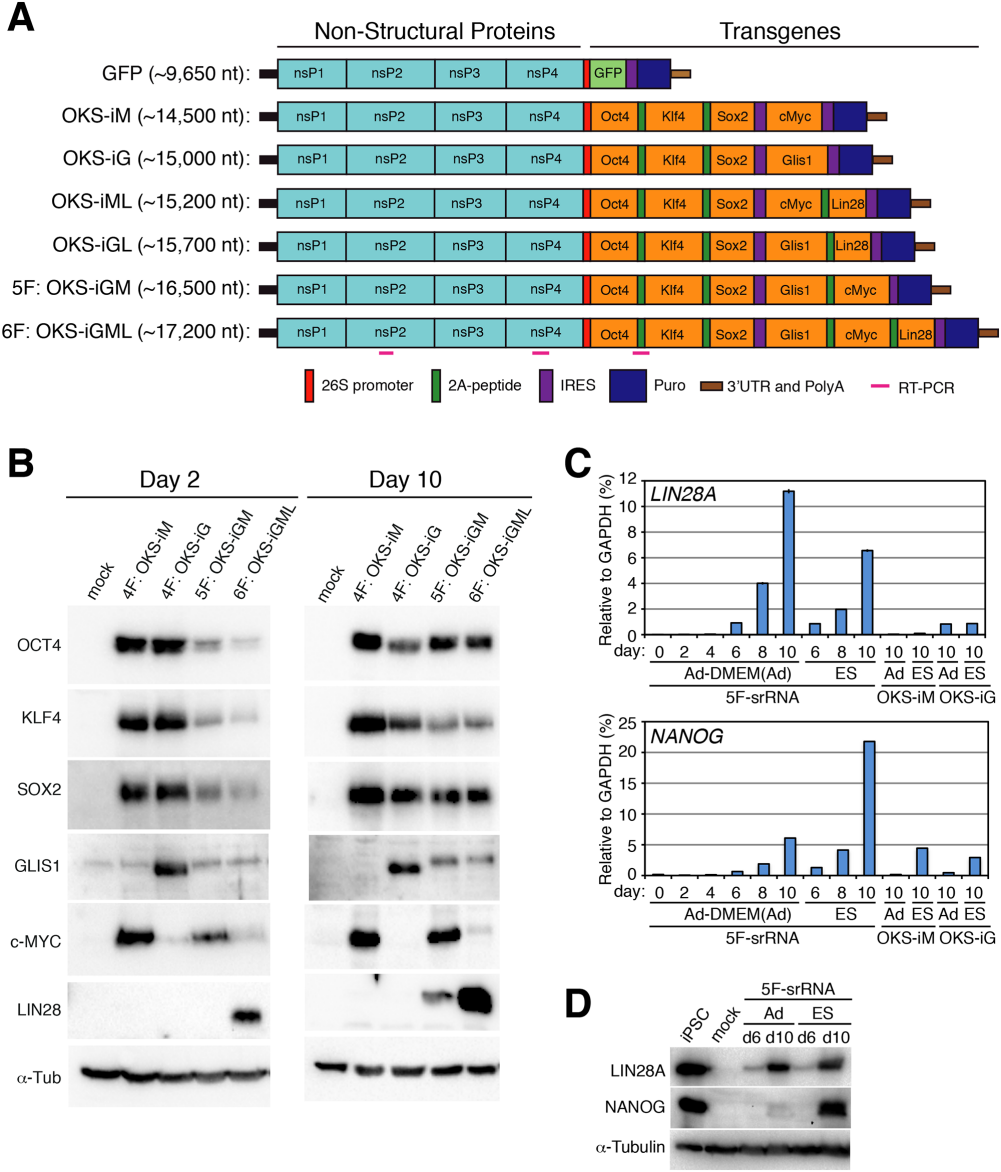
Construction and expression of srRNA. A: Scheme of Self-Replicating srRNA constructs. B: Expression of reprogramming factors in OKS-iM 4F-srRNA, OKS-iG 4F-srRNA, OKS-iGM 5F-srRNA, and OKS-iGML 6F-srRNA on day 2 (left) and day 10 (right). BJ cells were co-transfected with srRNAs plus B18R mRNA (1:1 srRNA:B18R mRNA), selected with puromycin and cultured in Advanced DMEM/10% FBS for 10 days. C: qRT-PCR analysis of *LIN28A* and *NANOG* from 4F-srRNA and 5F-srRNA transfected BJ cells normalized to GAPDH. Cells were selected with puromycin after the transfection, and cultured in Advanced DMEM/10% FBS (Ad) or ES medium. Cells were collected on day 2, 4, 6, 8 and 10 for 5F-srRNA, and day 10 for 4F-srRNA (OKS-iM and OKS-iG). D: Immunoblot analysis of LIN28A and NANOG in 5F-srRNA transfected BJ cells on day 6 and 10. iPSC: iPSC clone generated with 5F-srRNA from BJ cells.

We previously generated human iPSCs by a single transfection of 4F-srRNA (OKS-iM or OKS-iG) into neonatal and adult human fibroblasts [11]. However, the efficiency of iPSC colonies varied largely between high yield young human fibroblasts and older adult fibroblasts. To optimize iPSC colony yields regardless of cell source, we sought to improve 4F-srRNA by adding more reprogramming factor(s). Although many factors have been shown to reprogram fibroblasts, *LIN28A* is frequently used to increase the efficiency of iPSC generation by mRNAs and episomal plasmids in combination with the Yamanaka Factors (*OCT4*, *SOX2*, *KLF4*, and *cMYC*) [14–18]. LIN28A is highly expressed in undifferentiated cells and is an inhibitor of the let-7 miRNA that is induced after differentiation of ESC cells. Therefore, we added the *LIN28A* Open Reading Frame (ORF) to the 4F-srRNA construct after *cMYC* or *GLIS1* with an intervening self-cleavable P2A peptide to generate OKS-iML and OKS-iGL srRNAs, respectively (Fig 1A). The OKS-iML and OKS-iGL srRNAs were synthesized *in vitro* by T7 RNA polymerase, 5' capped, poly-Adenylated and transfected into two neonatal human fibroblasts (BJ and HFF) to generate iPSCs. To our surprise, we did not observe a significant increase in Alkaline Phosphatase (AP) positive colonies with the OKS-iML srRNA compared to 4F-srRNAs with only a <2-fold increase (S1 Fig). However, we noted that the OKS-iGL srRNA transfected cells produced many AP positive single cells that did not resolve into colonies, suggesting that the addition of *LIN28A* to 4F-srRNA resulted in partially reprogrammed cells.

One of let-7's direct targets is *cMYC*, a master transcription factor that drives cell division and metabolism [15, 17]. We hypothesized that LIN28A expression from OKS-iGL srRNA may enhance cMYC expression resulting in increased AP positive cell numbers and iPSC colonies. We cloned *cMYC* and *cMYC* plus *LIN28A* with an intervening self-cleavable P2A peptide into the OKS-iG srRNA to generate OKS-iGM 5F-srRNA and OKS-iGML 6F-srRNA, respectively (Fig 1A). Transfection of 5F-srRNA and 6F-srRNA into BJ fibroblasts resulted in a marked decrease in expression of all srRNA encoded reprogramming factors at day 2 compared to the parental 4F-srRNA (Fig 1B), likely due to increased length of the 5F- and 6F-srRNAs and a correspondingly decreased transfection efficiency. However, by day 10, we observed increased comparable expression of all genes, except for cMYC in 6F-srRNA transfected cells. To our surprise, LIN28A protein was measurably induced in 5F-srRNA transfected cells on day 10 (Fig 1B). qRT-PCR for *LIN28A* on days 2, 4, 6, 8 and 10 in 5F-srRNA transfected cells showed an increased expression starting on day 6 in both normal and ES culture medium (Fig 1C). However, we note that NANOG protein was strongly induced in ES culture medium in cells transfected with 5F-srRNA on day 10, but only weakly expressed in cells grown in normal media (Fig 1D).

### Comparison of iPSC generation with 4F, 5F and 6F-srRNAs

**Fig 2:**
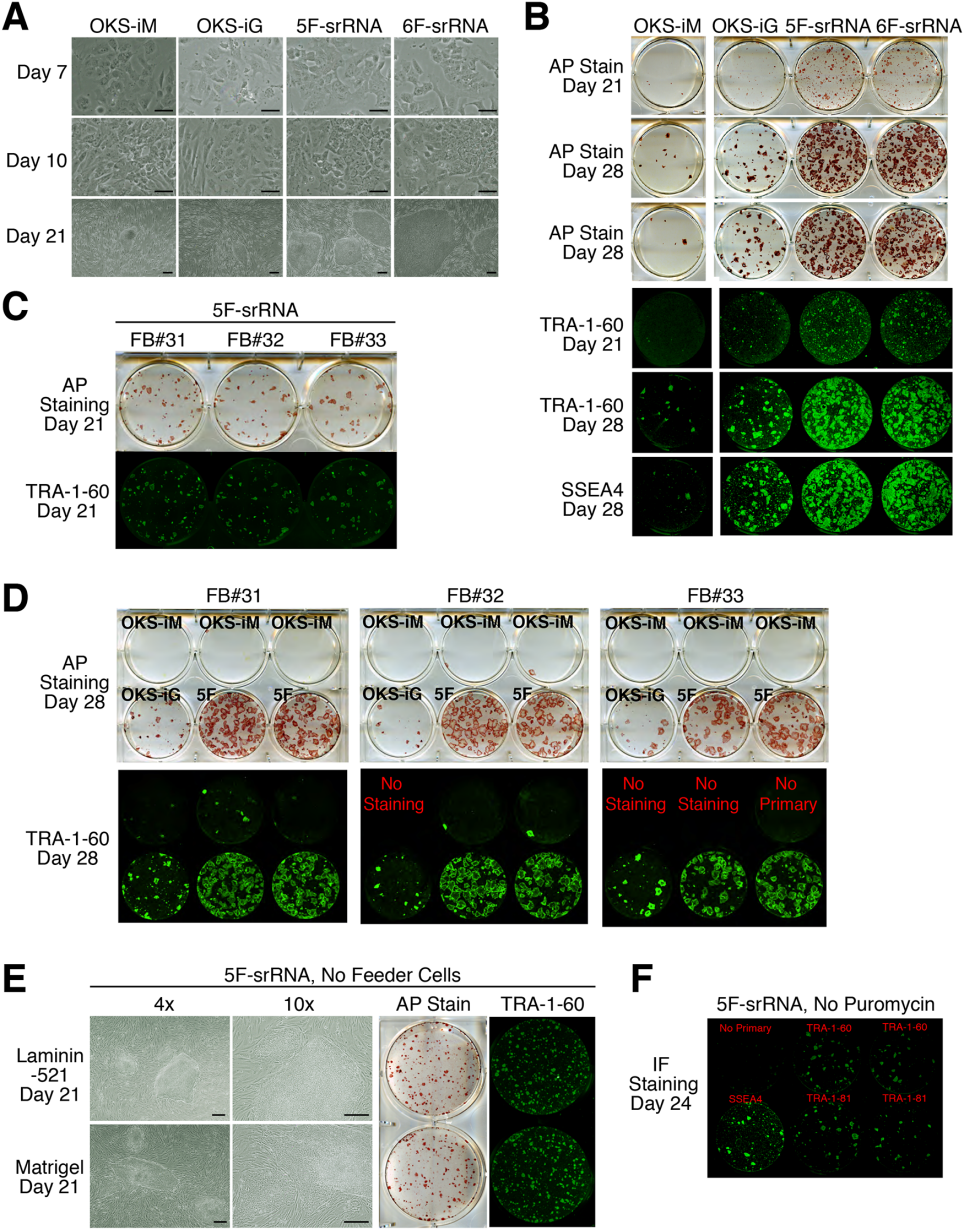
Comparison of iPSC generation with 4F-srRNA, 5F-srRNA and 6F-srRNA. A: iPSC generation in BJ cells. Cell images on day 7, 10, and 21. ES culture medium was used starting on day 7. Scale bar, 100 μm. B: iPSC generation in BJ cells. iPSC colonies were stained with Alkaline Phosphatase (AP) on day 21 and 28. TRA-1-60 and SSEA4 staining was performed after AP staining. On day 10, a starting well was passaged to 3 wells for OKS-iM, 2 wells for OKS-iG and 5F-srRNA. C: Day 21 iPSC colonies from adult human fibroblasts, FB#31, FB#32 and FB#33, from 5F-srRNA were stained with AP and TRA-1-60. FB#31, 55 year old healthy male donor, FB#32: 54 year old healthy female donor, FB#33: 24 year old cardiomyopathy male donor. D: Day 28 iPSC colonies from adult human fibroblasts, FB#31, FB#32 and FB#33, were stained with AP and TRA-1-60. A starting well was passaged to 3 wells for OKS-iM, 1 well for OKS-iG and 2 wells for 5F-srRNA. E: iPSC generation in feeder-free conditions. iPSC colonies were generated on Laminin- 521 or Matrigel. BJ cells were co-transfected with 5F-srRNA plus B18R mRNA (1:1 ratio), and selected with puromycin, then passaged onto Laminin-521 or Matrigel. iPSC colonies were stained with AP and TRA-1-60 on day 21. Scale bar, 100 μm. F: iPSC generation without puromycin selection. BJ cells were co-transfected with 5F-srRNA plus B18R mRNA (1:1 ratio), then on day 5, one well was passaged into 6 wells. AP staining and TRA-1-60, TRA-1-80, or SSEA4 staining were performed on day 24. The numbers of AP positive colonies per starting well were counted and are summarized in Tables 1 and 2.

We next compared the iPSC generation potential of 4F-srRNA (OKSiM and OKS-iG), 5F-srRNA (OKS-iGM), and 6F-srRNA (OKS-iGML). For iPSCs generation, BJ fibroblasts were transfected with each srRNA once, placed under puromycin selection and then passaged onto feeder cells. By day 7, both 5F-srRNA and 6F-srRNA transfected cells showed strong morphological changes, while only a small number of 4F-srRNA transfected cells showed signs of morphological change (Fig 2A). By day 10, both 5F-srRNA and 6F-srRNA transfected cells showed similar morphology of feeder-free human iPSCs in culture, namely round cells with large nuclei and clear nucleoli (Fig 2A). Consistent with the similarity of ESCs/iPSCs morphology, by day 15, visible iPSC colonies were present with most colonies large enough to isolate by day 21, a week earlier than that of 4F-srRNA transfected cells (Fig 2A). The number of 5F-srRNA and 6F-srRNA AP positive colonies was several fold higher than that of the 4F-srRNA (Fig 2B, Table 1, S1 Table). We did not observe any significant differences in iPSC colony numbers between 5F-srRNA and 6F-srRNA (Fig 2B, Table 1, S1 Table). In addition, most colonies from 4F-srRNA, 5F-srRNA and 6F-srRNA were positive for both TRA-1-60 and SSEA4 ES markers (Fig 2B). However, in contrast to the OKS-iGL srRNA, we observed very few partially reprogrammed cells with 5F-srRNA OKS-iGM and 6F-srRNA OKS-iGML. Taken together, both 5F-srRNA and 6F-srRNA efficiently generated iPSC colonies from young human BJ fibroblasts.

**Table 1:** srRNA generation of iPS cells from neonatal human fibroblasts.

	srRNA	Cell	Transfection Condition	Other Condition	Day of Passage	Number of AP+ Colonies	Day of AP Staining
Exp. 1	4F OKS-iM	BJ	1 μg srRNA	-	Day 10	15	Day 21
Exp. 1	4F OKS-iM	BJ	1 μg srRNA	-	Day 10	28	Day 28
Exp. 1	4F OKS-iG	BJ	1 μg srRNA	-	Day 10	83	Day 21
Exp. 1	4F OKS-iG	BJ	1 μg srRNA	-	Day 10	132	Day 28
Exp. 1	5F OKS-iGM	BJ	1 μg srRNA	-	Day 10	419	Day 21
Exp. 1	5F OKS-iGM	BJ	1 μg srRNA	-	Day 10	409	Day 28
Exp. 1	6F OKS-iGML	BJ	1 μg srRNA	-	Day 10	284	Day 21
Exp. 1	6F OKS-iGML	BJ	1 μg srRNA	-	Day 10	390	Day 28
Exp. 2	5F OKS-iGM	BJ	1 μg srRNA	-	Day 10	522	Day 21
Exp. 2	5F OKS-iGM	BJ	1 μg srRNA	Laminin-521	Day 10	760	Day 21
Exp. 2	5F OKS-iGM	BJ	1 μg srRNA	Matrigel	Day 10	656	Day 21
Exp. 3	5F OKS-iGM	BJ	1 μg srRNA	No puro	Day 5	124	Day 24
Exp. 3	5F OKS-iGM	BJ	2 μg srRNA	No puro	Day 5	187	Day 24
Exp. 3	5F OKS-iGM	BJ	4 μg srRNA	No puro	Day 5	168	Day 24
Exp. 3	5F OKS-iGM	BJ	6 μg srRNA	No puro	Day 5	207	Day 24
Exp. 6	5F OKS-iGM	BJ	1 μg srRNA	Passage 21	Day 10	76	Day 29
Exp. 6	5F OKS-iGM	HFF	1 μg srRNA	Passage 30	Day 10	149	Day 29

Cells were passaged on 6-well plate on day 0 and cultured to 80–100% confluency on day 1. To minimize the IFN responses, cells were treated with 20% B18R-CM 20 min before the transfection. The srRNA was transfected with Lipofectamine 2000 in the presence of 20% B18R-CM (No B18R mRNA co-transfection). Medium was changed to the Advanced DMEM containing 20% B18R-CM after 3hr transfection. ES culture medium was used from day 7. Cells were passaged on feeder cells as indicated day except for Exp.2 (feeder free condition). Passage 8 to 10 of BJ cells was used except for Exp.6. Colonies were stained with Alkaline Phosphatase (AP) and the numbers of AP positive colonies per starting well were indicated.

We next compared the generation of iPSC colonies by 4F-srRNA, 5F-srRNA and 6F-srRNA with three adult human fibroblasts. FB#31 was a healthy 55 year old male donor, FB#32 was a healthy 54 year old female donor, and FB#33 was a 24b year old male cardiomyopathy patient donor. Similar to BJ cells, many large iPSC colonies were generated by 5F-srRNA transfection on day 21 (Fig 2C). By day 28, we observed several fold more AP positive and TRA-1-60 positive iPSC colonies with 5F-srRNA than 4F srRNAs in all three older fibroblasts (Fig 2D). Although FB#33 fibroblasts were derived from a donor with cardiomyopathy disease, the number of iPSC colonies was comparable with older healthy donor fibroblasts FB#31 and FB#32. We also assayed 5F-srRNA in three additional adult human fibroblasts, HFB (female, age 77), HDF (male, age 56) and NHDFc (female, age 50), which resulted in the efficient generation of iPSC colonies (Table 2). Moreover, 5F-srRNA also generated many iPSC colonies in high passage neonatal fibroblasts, BJ passage 21 and HFF passage 30 (Table 1).

**Table 2:** srRNA generation of iPS cells from adult human fibroblasts.

RNA Replicon	Cell name/yrs/sex	Transfection condition	Number of AP+ Colonies	Day of AP staining
4F OKS-iM	FB#31/55yrs/m	1 μg of RNAs	1	Day 28
4F OKS-iG	FB#31	1 μg of RNAs	64	Day 28
5F OKS-iGM	FB#31	1 μg of RNAs	207	Day 21
5F OKS-iGM	FB#31	1 μg of RNAs	275	Day 28
4F OKS-iM	FB#32/54yrs/f	1 μg of RNAs	2	Day 28
4F OKS-iG	FB#32	1 μg of RNAs	16	Day 28
5F OKS-iGM	FB#32	1 μg of RNAs	141	Day 21
5F OKS-iGM	FB#32	1 μg of RNAs	203	Day 28
4F OKS-iM	FB#33/24yrs/m	1 μg of RNAs	0	Day 28
4F OKS-iG	FB#33	1 μg of RNAs	30	Day 28
5F OKS-iGM	FB#33	1 μg of RNAs	123	Day 21
5F OKS-iGM	FB#33	1 μg of RNAs	180	Day 28
4F OKS-iM	NHDFc/50yrs/f	1 μg of RNAs	5	Day 23
4F OKS-iG	NHDFc	1 μg of RNAs	110	Day 23
5F OKS-iGM	NHDFc	1 μg of RNAs	281	Day 23
5F OKS-iGM	HFB/77yrs/f	1 μg of srRNA	557	Day 20
5F OKS-iGM	HDF/56yrs/m	1 μg of srRNA	146	Day 29

Cells were passaged on 6-well pate on day 0 and cultured to 80–100% confluency on day 1. Cells were pretreated with 20% B18R-CM prior to transfection. For FB#31, #32, #33 and NHDFc, cells were co-transfected with srRNA plus B18R-mRNA (1:1 ratio, total 1 μg RNA/well). For HFB and HDF, cells were transfected with srRNA without B18R-mRNA. After 3 hr, medium was changed to the Advanced DMEM containing 20% B18R-CM. Puromycin selection (0.8 μg/well) was performed from day 2 to 10. ES medium was used starting on day 7, and cells were passaged onto feeder cells on day 10. Colonies were stained with Alkaline Phosphatase (AP) and numbers of AP positive colonies per starting well were indicated.

Given the efficiency of 5F-srRNA iPSC to generate iPSCs, we tested 5F-srRNA under feeder-free conditions. We observed many iPSC colonies in 5F-srRNA feeder-free conditions using Laminin-521 or Matrigel (Fig 2E, Table 1). We also examined iPSC generation with 5F-srRNA without puromycin selection. Due to cell density under this condition, we passaged one well to six wells on day 5. Although the no puromycin selection condition decreased the overall number of iPSC colonies, we detected more than 100 iPSC colonies from a single starting well (Fig 2F, Table 1, S1 Table). Increasing the 5F-srRNA from 1 to 2, 4 and 6 μg per well (6-well plate scale) slightly increased the number of iPSC colonies, but we also increased some cytotoxicity after the 4 and 6 μg transfections.

### Characterization of iPSC clones from 5F-srRNA

**Fig 3:**
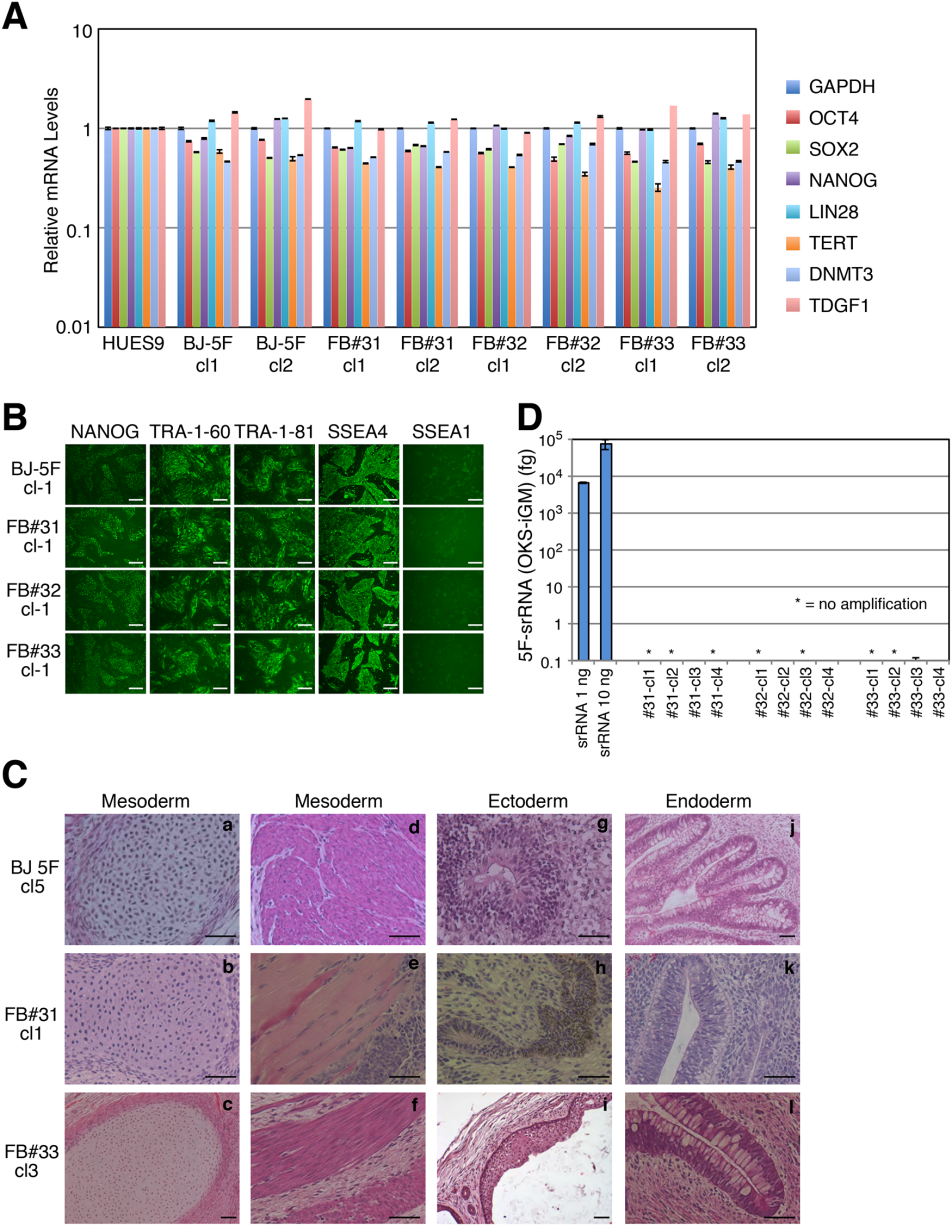
Characterization of iPSC clones from 5F-srRNA. A: qRT-PCR analysis of ES cells markers on 5F-srRNA iPSC clones. Expression of ES marker genes were normalized to GAPDH. B: Immunofluorescence analysis of ES markers in 5F-srRNA iPSC clones from BJ, FB#31, FB#32 and FB#33. Scale bar, 100 mm. C: Teratoma formation of 5F-srRNA iPSC clones. iPSC clones were injected intramuscular or subcutaneous into NRG mice. Teratomas were allowed to form for 4–6 months after injection. Four of five BJ-5F-iPSC clones (#1, 3, 4, 5), three of three FB#31-5F-iPSC clones (#1, 2, 3), and one of two FB#33-5F-iPSC clones (#3) generated teratomas. Cartilage (a, b, c), muscle (d, e, f), neural tissue (g), pigmented epithelium (h), squamous epithelium (i), intestinal epithelium (j, f), and respiratory epithelium (k). Scale bar, 50 μm. D: qRT-PCR for srRNA (nsP1 region) was performed on FB#31, FB#32, and FB #33 -5F-srRNA iPSC clones. Total RNA was isolated from passage 5 (P5) cells. * = no detected amplification.

To characterize iPSCs generated by 5F-srRNA, we isolated multiple colonies from 5F-srRNA transfections of BJ, FB#31, FB#32, and FB#33 fibroblasts. qRT-PCR analysis for expression of OCT4 (endogenous), SOX2 (endogenous), NANOG, LIN28A, TERT, DNMT3, and TDGF1 in each clone was comparable with HUES9 human ESCs (Fig 3A). In addition, all iPSC colonies were positive for NANOG, TRA-1-60, TRA-1-81 and SSEA4, and negative for SSEA1 by immunofluorescence staining (Fig 3B). We also evaluated the pluripotency of iPSC clones by teratoma formation in mice. Teratomas were generated from four of five clones from BJ 5F-srRNA iPSCs, three of three clones from FB#31 5F-srRNA iPSCs, and one of two clones from FB#33 5F-srRNA iPSCs (Fig 3C). We next examined if 5F-srRNA iPSC generated clones had become srRNA (Replicon) free. srRNA persists in cells grown in the presence of B18R protein, which binds to and neutralizes type I IFNs [13]. However, srRNA is quickly degraded in the absence of B18R protein [11]. Primary iPSC colonies were mechanically isolated and passaged in the absence of B18R. After Passage 3 (P3), cells were grown in 6-well plates and passaged 1:5 dilutions every 4 or 5 days. Most iPSC clones had lost the srRNA by P3 and all iPSC clones were negative by P5 by qRT-PCR (Fig 3D and S2 Fig).

### Improvement of 5F-srRNA quality and transfection efficiency

During the 5F-srRNA and 6F-srRNA experiments, we detected differences of srRNA quality that were due to the 5' capping reaction. We used an enzymatic capping method and 2’-O-methyltransferase that generates a Cap-1 RNA with a theoretical yield near 100%. However, we observed varied transfection efficiencies depending on the capping reaction enzyme lot number from commercial venders. Poor capping efficiency leads to srRNAs contaminated with non-capped srRNA that contain a 5’-triphosphate that induces strong RIG-I and PKR innate immune responses [14, 19–22]. To circumvent this, after the capping reaction, we treated the srRNA with phosphatase to remove the 5’-triphosphate. Transfection of phosphatase treated 5F-srRNA resulted in an increased number of cells after puromycin selection and relieved the problem of srRNA quality derived from different lot number of capping enzymes (S3 Fig). Likewise, phosphatase treatment of GFP srRNA resulted in a dose-dependent increase of transfection efficiency and higher expression of GFP vs. non-phosphatase treated GFP srRNA (S3 Fig). These data suggest that phosphatase treatment increased the quality of srRNA by decreasing the IFN responses.

**Fig 4:**
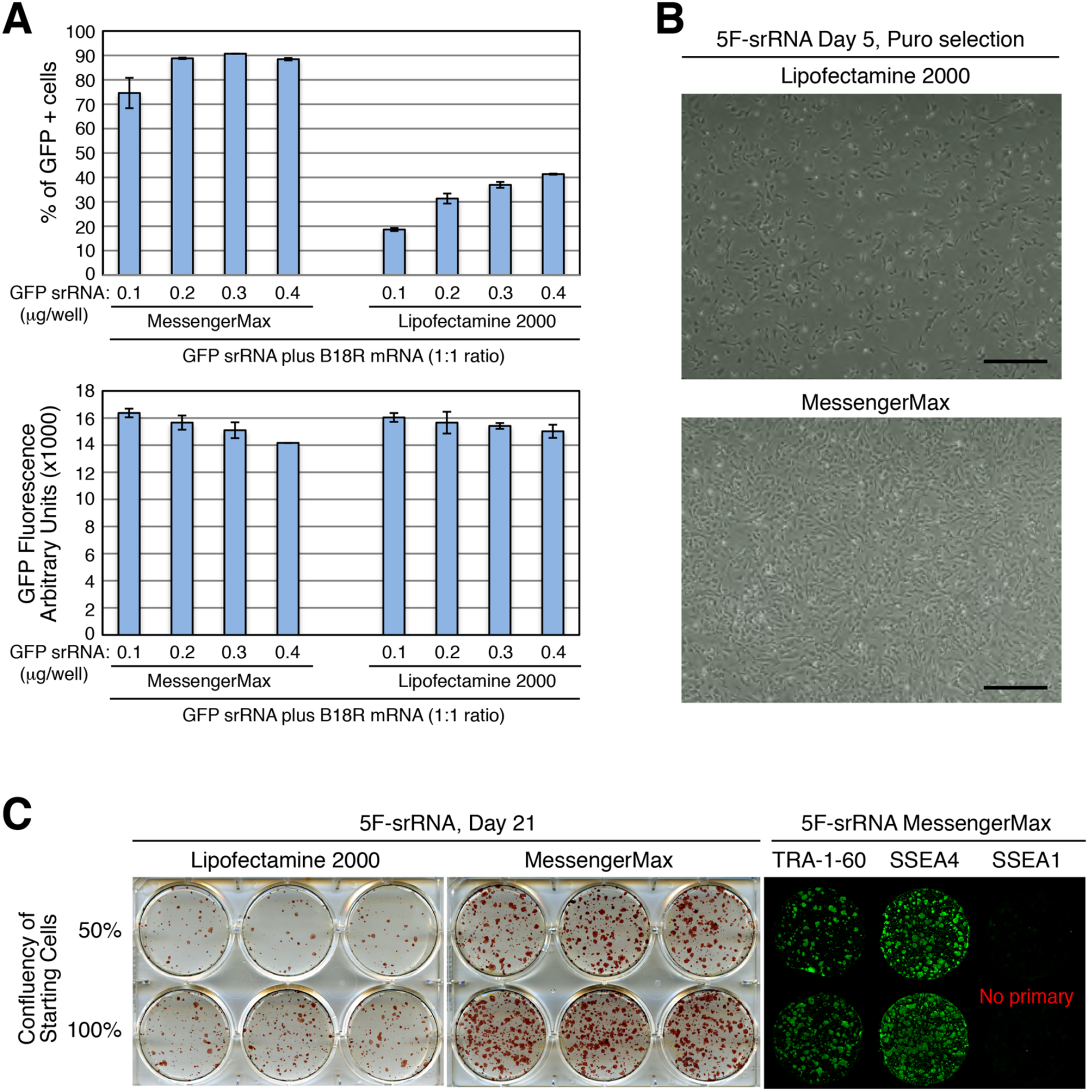
5F-srRNA iPSC generation with messengerMax transfection. A: Comparison of Lipofectamine 2000 and MessengerMax transfection efficiency. GFP srRNA was co-transfected with B18R mRNA (1: 1 ratio) into BJ cells and GFP expression was measured by flow cytometry on day 2. B: 5F-srRNA was co-transfected with B18R mRNA (1:1 ratio) into BJ cells, and selected for puromycin for 4 days (Day 5). Scale bar, 200 μm. C: Comparison of iPSC generation with Lipofectamine-2000 and MessengerMax transfection. iPSC colonies were generated, and stained with AP, TRA-1-60, SSEA4 and SSEA1, as indicated.

In our hands, the transfection efficiency of GFP srRNA (~10 kb) into human fibroblasts using Lipofectamine 2000 is 40~60%, while the transfection efficiency of GFP mRNA (~0.9 kb) is > 90%. To improve iPSC generation efficiency, we tested a newly released transfection reagent, Lipofectamine MessengerMAX. Using Lipofectamine MessengerMAX we obtained ~90% transfection efficiency of the 10 kb GFP srRNA (Fig 4A). Likewise, transfection of 5F-srRNA by Lipofectamine MessengerMAX into BJ cells resulted in an increased number of surviving cells compared to Lipofectamine 2000 transfection (Fig 4B). By day 21 post transfection of 5F-srRNA, we obtained several fold more iPSC colonies with Lipofectamine MessengerMAX vs. Lipofectamine 2000 transfection (Fig 4C, Table 3, S2 Table). After optimizing Lipofectamine MessengerMAX conditions, we obtained >400 iPSC colonies without puromycin selection and more than 100 iPSC colonies without puromycin selection in feeder-free conditions (Table 3, S2 Table).

**Table 3:** 5F-srRNA generation of iPSCs by messengerMAX transfection in BJ cells.

Transfection Reagent	Confluency of starting cells	Puro selection	Day of Passage	Passage plate	Passage dilution	Number of AP+ Colonies
mMAX	50%	+	Day 5	feeder	1 to 3	556
mMAX	100%	+	Day 5	feeder	1 to 3	820
mMAX	50%	+	Day 7	feeder	1 to 3	703
mMAX	100%	+	Day 7	feeder	1 to 3	963
L2K	50%	+	Day 7	feeder	1 to 3	219
L2K	100%	+	Day 7	feeder	1 to 3	449
mMAX	50%	-	Day 3	feeder	1 to 6	571
mMAX	100%	-	Day 3	feeder	1 to 6	730
mMAX	50%	-	Day 5	feeder	1 to 6	416
mMAX	100%	-	Day 5	feeder	1 to 6	681
mMAX	50%	-	Day 3	feeder	1 to 12	660
mMAX	50%	-	Day 3	Matrigel	1 to 12	46
mMAX	50%	-	Day 5	feeder	1 to 12	514
mMAX	50%	-	Day 5	Matrigel	1 to 12	112

BJ cells (Passage 8) were plated on 6-well on day 0 and cultured to 50 or 100% confluency on day 1. Cells were co-transfected with 5F srRNA plus B18R mRNA (1:1 ratio, 2 μg RNA/well) in the absence of serum and B18R-CM. After 3 hr, medium was changed to the Advanced DMEM containing 20% B18R-CM. ES culture medium was used starting on day 5. Cells were passaged onto feeder cells or Matrigel as indicated. Colonies were stained with Alkaline Phosphatase (AP) on day 21 and numbers of AP positive colonies per starting well are reported. mMAX = MessengerMAX transfection reagent; L2K = Lipofectamine 2000 transfection reagent.

## Discussion

Many integration-free methods, including episomal plasmids [16, 18], mRNAs transfection [14], Sendai virus [23], and srRNA [11], have been developed for the generation of human iPSCs [8, 9]. While Sendai viruses and srRNA method can reproduce the iPSC generation of retrovirus method(s) without additional reprogramming factor, episomal plasmids and mRNAs methods need additional reprogramming factor(s) to obtain iPSC colonies. Although the 4F-srRNA efficiently generated iPSCs from young human fibroblasts, the efficiency of iPSC generation for older adult human fibroblasts was not acceptable. Here we expanded on our 2013 report [11] with the specific goal of greatly enhancing the ability to induce iPSCs from old adult human fibroblasts.

To do so, we cloned in additional reprogramming factor(s) open-reading frames into the single srRNA and assayed for iPSC generation. We found that the five factor combination of *OCT4*, *KLF4*, *SOX2*, *GLIS1*, and *cMYC* was superior to both 6F-srRNA, that also contains *LIN-28A*, and our original 4F-srRNA (OKS-iM or OKS-iG) [11]. Previously, Maekawa et al. [24] reported a synergistic increase of iPSC generation by combination of OSKMG (*Oct4*, *Sox2*, *Klf4*, *cMyc* and *Glis1*) in mouse fibroblasts, but not in human fibroblasts, by introducing these factors with retrovirus infection. In human fibroblasts, they obtained similar iPSC colonies with OSKMG and OSKG, and both were better than OSKM. In contrast, 5F srRNA containing *OCT4*, *KLF4*, *SOX2*, *GLIS1*, and *cMYC* significantly increased the human iPSC generation as compared to both 4F-iM and 4F-iG srRNAs, especially in old adult human fibroblasts. Moreover, compared to retroviral infection with each reprogramming factor or transfection of individual mRNAs, 5F srRNA expresses each factor from a single polycistronic srRNA, so the ratio of reprogramming factors is constant in each cell. This is critical, because the expression ratio of reprogramming factors affects on the efficiency of human iPSC generation [25, 26]. We have previously reported that too high of levels of *cMYC* inhibit the generation of iPSCs [11]. We also observed a faster iPSC generation time with 5F-srRNA and 6F srRNAs compared to the 4F-srRNAs by 7 days, as well as larger sized iPSC colonies on day 21. Indeed, some iPSC colonies were ready to be isolated on day 15. Lastly, we also observed strong and early induction of LIN28A and NANOG in ES culture medium by 5F-srRNA. Together, these data support the synergistic effect of the five factor 5F-srRNA for human iPSC generation.

We tested the ability of 5F-srRNA to generate iPSCs on six different older adult human fibroblasts from 24 to 77 years of age and obtained more than 100 iPSC colonies from a starting well (6-well plate scale). A parallel comparison of 4F-srRNA and 5F-srRNA showed that 5F-srRNA generated iPSC colonies 4-fold to >10-fold more efficiently than the 4F-srRNA (Table 2). Moreover, FB#33 fibroblasts are derived from a cardiomyopathy patient and generated iPSC colonies comparably to healthy donor’s fibroblasts. The vast majority of iPSC colonies from 5F-srRNA were AP positive and TRA-1-60, TRA-1-80 or SSEA4 positive. Therefore, we believe that the 5F-srRNA overcomes several problems that the 4F-srRNA was unable to for generating iPSCs, including the high efficiency of iPSC generation with all sources and age of adult human fibroblasts.

Lastly, during preliminarily experiments, we detected a low capping efficiency of srRNAs resulting in activation of an innate immune response. However, the addition of a phosphatase treatment step to remove the 5’-triphosphate of non-capped RNA successfully removed the non-capped RNA and solved this problem. Furthermore, because we observed some srRNA degradation during the poly A reaction step (data not shown), we built in a 150 base poly A tail into backbone srRNA vector to omit the poly A tail reaction. This improvement increased the srRNA quality, and resulted in a slight increase in the number of iPSC colonies (data not shown). Previously due to a poor transfection of the 16.5 kb 5F-srRNA, we performed a puromycin selection step to remove untransfected cells. However, using the new Lipofectamine MessengerMAX transfection reagent for long RNAs sufficiently increased the transfection efficiency and thereby allowed us to eliminate the puromycin selection. Notably we obtained similar number of iPSC colonies comparable to 5F-srRNA transfection with puromycin selection.

In summary, a single transfection of 5F-srRNA efficiently generated iPSC colonies from six different adult human fibroblasts ranging from 24 to 77 years old, including a cardiomyopathy patient donor cells, seven days faster than our 4F-srRNA [11]. Surprisingly, 5F-srRNA induced endogenous LIN28A and NANOG expression that likely further enhanced and helped to accelerate iPSC generation by a week. We believe that 5F-srRNA method represents a significant improvement in the generation iPSCs, especially when using older adult fibroblasts.

## Conclusion

We generated a 5F-srRNA that worked faster and efficiently for generating iPSCs in all human fibroblasts tested including a cardiomyopathy patient donor. Moreover, by improving srRNA quality with phosphatase treatment and using a new transfection reagent, Lipofectamine MessengerMAX, a single 5F-srRNA transfection resulted in ~90% efficiency of iPSC generation. Together, these improvements enabled the generation of iPSCs without the need for puromycin selection or feeder cells.

## Supporting information

S1 Fig Addition of Lin28A into OKS-iG increased the iPSC colony numbers. **A** iPSC generation byh OKS-IM, OKS-iG, OKS-iML and OKS-iGL srRNAs in BJ cells. iPSCs were generated by co-transfection of srRNA plus B18R mRNA (1:1 ratio) in BJ and HFF cells. AP staining of iPS colonies performed on day 28. **B** Number of AP positive iPSC colonies per starting 6 well.
(PDF)

S2 Fig qRT-PCR analysis of BJ 5F-srRNA iPSC clones. **A** qRT-PCR analysis of 5F-srRNA in BJ-5F-srRNA iPSC clones. "-" = BJ cells without sr-RNA (negative control); "+" = srRNA transfected BJ cells (positive control). BJ-5F-clones (1–8). iPSC clones were mechanically isolated and cultured in the absence of B18R. Total RNA was isolated from Passage 3 and 5, and used for qRT-PCR. Primers were in nsP2 region and Oct4-Klf4 (OK) region. **B** qRT-PCR analysis with TaqMan probe for srRNA (nsP1 region) was performed in BJ-5F-srRNA clones (1–8). * = no amplification.
(PDF)

S3 Fig Phosphatase treatment improves srRNA transfection efficiency. **A** OCT4 expression with phosphatase treated 5F-sRNA. 5F-srRNA was prepared with or without phosphatase treatment. 5F-srRNA and B18R mRNA were co-transfected into BJ cells with Lipofectamine 2000 for Western blotting. 5F-Phos (-): no phosphatase treated 5F-srRNA; 5F-Phos(+): phosphatase treated 5F-srRNA. **B** TagGFP2 srRNA (gifted from EMD Millipore) was prepared with or without phosphatase treatment. TagGFP2 srRNA was transfected into BJ cells with MessengerMax reagent in the presence of recombinant B18R protein. One day after the transfection, GFP expression was measured by FACS. Phos (+): Phosphatase treated srRNA; Phos (-): No phosphatase treated srRNA. **C** Microphotographs of cells from (A). Scale bar, 250 μm.
(PDF)

S1 Table Oligonucleotides used for plasmid constructs. (PDF)

S2 Table srRNA generation of iPS cells from neonatal human fibroblasts. (PDF)

S3 Table 5F-srRNA generation of iPSCs by messengerMAX transfection in BJ cells. (PDF)

## References

[1] Takahashi K, Yamanaka S. Induction of pluripotent stem cells from mouse embryonic and adult fibroblast cultures by defined factors. Cell. 2006;126: 663–676. doi: 10.1016/j.cell.2006.07.024 16904174 10.1016/j.cell.2006.07.024

[2] Takahashi K, Tanabe K, Ohnuki M, Narita M, Ichisaka T, Tomoda K, et al Induction of pluripotent stem cells from adult human fibroblasts by defined factors. Cell. 2007;131:861–872. doi: 10.1016/j.cell.2007.11.019 18035408 10.1016/j.cell.2007.11.019

[3] Yu J, Vodyanik MA, Smuga-Otto K, Antosiewicz-Bourget J, Frane JL, Tian S, et al Induced pluripotent stem cell lines derived from human somatic cells. Science. 2007;318: 1917–1920. doi: 10.1126/science.1151526 18029452 10.1126/science.1151526

[4] Ben-David U, Benvenisty N. The tumorigenicity of human embryonic and induced pluripotent stem cells. Nat Rev Cancer. 2011;11: 268–277. doi: 10.1038/nrc3034 21390058 10.1038/nrc3034

[5] González F, Boué S, Izpisúa Belmonte JC. Methods for making induced pluripotent stem cells: reprogramming à la carte. Nat Rev Genet. 2011;12: 231–242. doi: 10.1038/nrg2937 21339765 10.1038/nrg2937

[6] Hussein SM, Nagy K, Nagy A. Human induced pluripotent stem cells: the past, present, and future. Clin Pharmacol Ther. 2011;89: 741–755. doi: 10.1038/clpt.2011.37 21430659 10.1038/clpt.2011.37

[7] Okita K, Ichisaka T, Yamanaka S. Generation of germline-competent induced pluripotent stem cells. Nature. 2007;448: 313–317. doi: 10.1038/nature05934 17554338 10.1038/nature05934

[8] Alateeq S, Fortuna PR, Wolvetang E. Advances in reprogramming to pluripotency. Curr Stem Cell Res Ther. 2015:10: 193–207. 25697500 10.2174/1574888x10666150220154820

[9] Hu K. All roads lead to induced pluripotent stem cells: the technologies of iPSC generation. Stem Cells Dev. 2014;23: 1285–1300. doi: 10.1089/scd.2013.0620 24524728 10.1089/scd.2013.0620 PMC4046204

[10] Schlaeger TM, Daheron L, Brickler TR, Entwisle S, Chan K, Cianci A, et al A comparison of non-integrating reprogramming methods. Nat Biotech. 2015:33: 58–63. 10.1038/nbt.3070 PMC4329913 25437882

[11] Yoshioka N, Gros E, Li HR, Kumar S, Deacon DC, Maron C, et al Efficient generation of human iPSCs by a synthetic self-replicative RNA. Cell Stem Cell. 2013;13: 246–254. doi: 10.1016/j.stem.2013.06.001 23910086 10.1016/j.stem.2013.06.001 PMC3845961

[12] Kinney RM, Johnson BJ, Welch JB, Tsuchiya KR, Trent DW. The full-length nucleotide sequences of the virulent Trinidad donkey strain of Venezuelan equine encephalitis virus and its attenuated vaccine derivative, strain TC-83. Virology. 1989;170: 19–30. 2524126 10.1016/0042-6822(89)90347-4

[13] Alcamí A, Symons JA, Smith GL. The vaccinia virus soluble alpha/beta interferon (IFN) receptor binds to the cell surface and protects cells from the antiviral effects of IFN. J Virol. 2000;74: 11230–11239. 11070021 10.1128/jvi.74.23.11230-11239.2000 PMC113220

[14] Warren L, Manos PD, Ahfeldt T, Loh YH, Li H, Lau F, et al Highly efficient reprogramming to pluripotency and directed differentiation of human cells with synthetic modified mRNA. Cell Stem Cell. 2010;7: 618–630. doi: 10.1016/j.stem.2010.08.012 20888316 10.1016/j.stem.2010.08.012 PMC3656821

[15] Mayr F, Heinemann U. Mechanisms of Lin28-mediated miRNA and mRNA regulation—a structural and functional perspective. Int J Mol Sci. 2013;14: 16532–16553. doi: 10.3390/ijms140816532 23939427 10.3390/ijms140816532 PMC3759924

[16] Okita K, Yamakawa T, Matsumura Y, Sato Y, Amano N, Watanabe A, et al An efficient nonviral method to generate integration-free human-induced pluripotent stem cells from cord blood and peripheral blood cells. Stem Cells. 2013;31: 458–466. doi: 10.1002/stem.1293 23193063 10.1002/stem.1293

[17] Rosa A, Brivanlou AH. Regulatory non-coding RNAs in pluripotent stem cells. Int J Mol Sci. 2013;14: 14346–14373. doi: 10.3390/ijms140714346 23852015 10.3390/ijms140714346 PMC3742248

[18] Yu J, Hu K, Smuga-Otto K, Tian S, Stewart R, Slukvin II, et al Human induced pluripotent stem cells free of vector and transgene sequences. Science. 2009;324: 797–801. doi: 10.1126/science.1172482 19325077 10.1126/science.1172482 PMC2758053

[19] Angel M, Yanik MF. Innate immune suppression enables frequent transfection with RNA encoding reprogramming proteins. PLoS One. 2010;5(7): e11756 doi: 10.1371/journal.pone.0011756 20668695 10.1371/journal.pone.0011756 PMC2909252

[20] Hornung V, Ellegast J, Kim S, Sato Y, Amano N, Watanabe A, et al 5'-Triphosphate RNA is the ligand for RIG-I. Science. 2006;314: 994–997. doi: 10.1126/science.1132505 17038590 10.1126/science.1132505

[21] Nallagatla SR, Toroney R, Bevilacqua PC. A brilliant disguise for self RNA: 5'-end and internal modifications of primary transcripts suppress elements of innate immunity. RNA Biol. 2008;5: 140–144. 18769134 10.4161/rna.5.3.6839 PMC2809118

[22] Pichlmair A, Schulz O, Tan CP, Näslund TI, Liljeström P, Weber F, et al RIG-I-mediated antiviral responses to single-stranded RNA bearing 5'-phosphates. Science. 2006; 314:997–1001. doi: 10.1126/science.1132998 17038589 10.1126/science.1132998

[23] Fusaki N, Ban H, Nishiyama A, Saeki K, Hasegawa M. Efficient induction of transgene-free human pluripotent stem cells using a vector based on Sendai virus, an RNA virus that does not integrate into the host genome. Proc Jpn Acad Ser B Phys Biol Sci. 2009;85: 348–362. doi: 10.2183/pjab.85.348 19838014 10.2183/pjab.85.348 PMC3621571

[24] Maekawa M, Yamaguchi K, Nakamura T, Shibukawa R, Kodanaka I, Ichisaka T, et al Direct reprogramming of somatic cells is promoted by maternal transcription factor Glis1. Nature. 2011;474: 225–229. doi: 10.1038/nature10106 21654807 10.1038/nature10106

[25] Liu X, Sun H, Qi J, Wang L, He S, Liu J, et al Sequential introduction of reprogramming factors reveals a time-sensitive requirement for individual factors and a sequential EMT-MET mechanism for optimal reprogramming. Nat Cell Biol. 2013;15: 829–838. doi: 10.1038/ncb2765 23708003 10.1038/ncb2765

[26] Papapetrou EP, Tomishima MJ, Chambers SM, Mica Y, Reed E, Menon J, et al Stoichiometric and temporal requirements of Oct4, Sox2, Klf4, and c-Myc expression for efficient human iPSC induction and differentiation. Proc Natl Acad Sci USA. 2009;106: 12759–12764. doi: 10.1073/pnas.0904825106 19549847 10.1073/pnas.0904825106 PMC2722286

