# Supplementary figures and images for: Enhanced generation of iPSCs from older adult human cells by a synthetic five-factor self-replicative RNA
Source: PLoS One. 2017 Jul 27;12(7):e0182018. doi: 10.1371/journal.pone.0182018 (PMC5531586; doi:10.1371/journal.pone.0182018)

Supporting Information Figure 1, Yoshioka & Dowdy

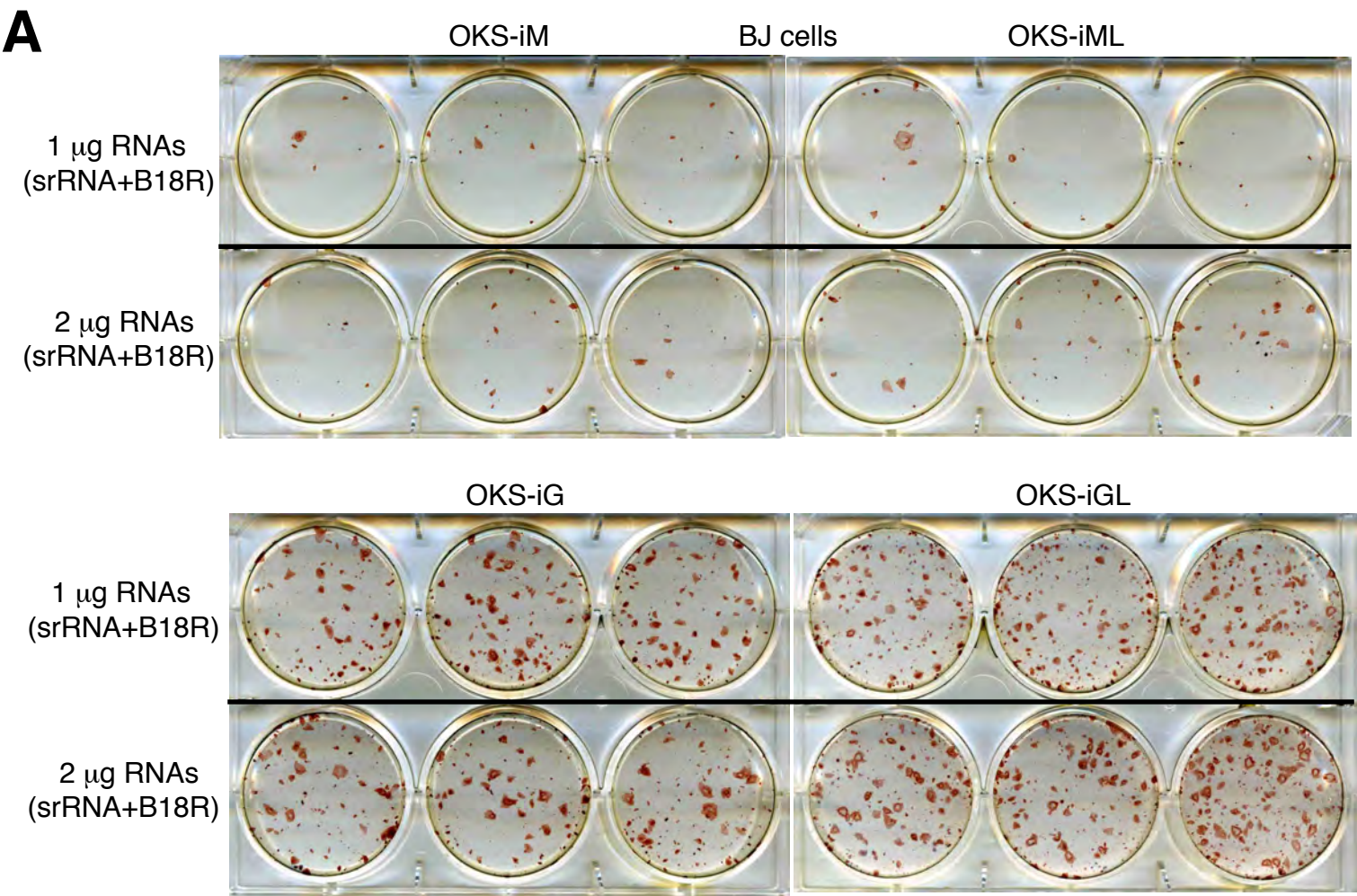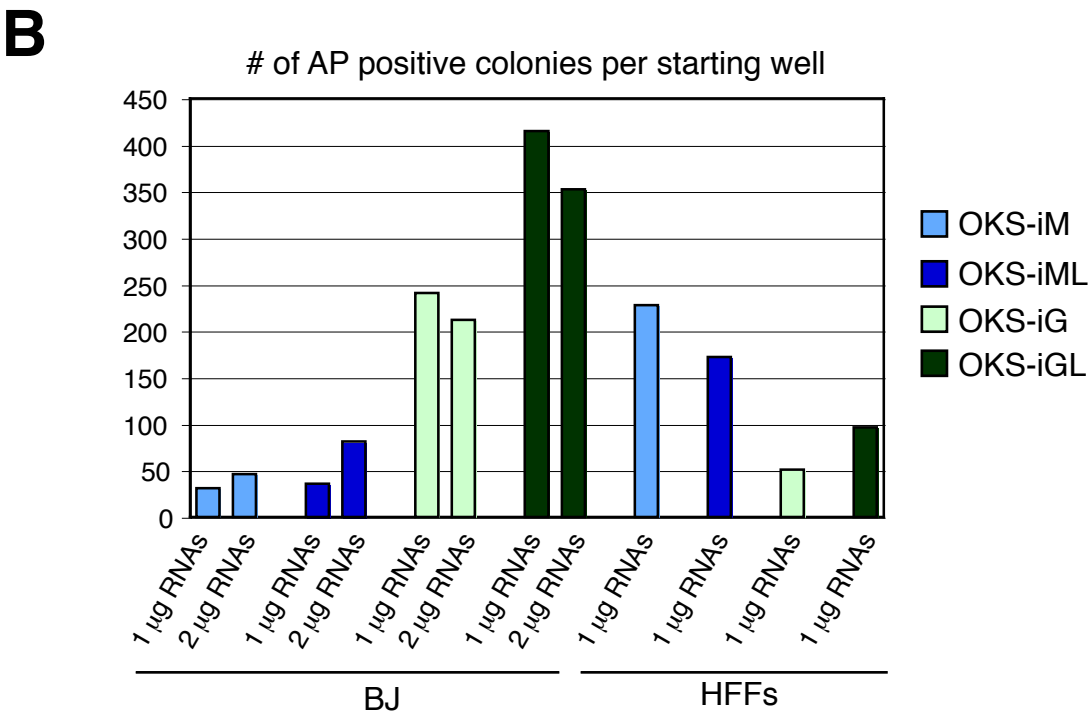

Supplement: S1 Fig — A iPSC generation byh OKS-IM, OKS-iG, OKS-iML and OKS-iGL srRNAs in BJ cells. iPSCs were generated by co-transfection of srRNA plus B18R mRNA (1:1 ratio) in BJ and HFF cells. AP staining of iPS colonies performed on day 28. B Number of AP positive iPSC colonies per starting 6 well. (PDF) [file pone.0182018.s001.pdf]

Supporting Information Figure 2, Yoshioka & Dowdy

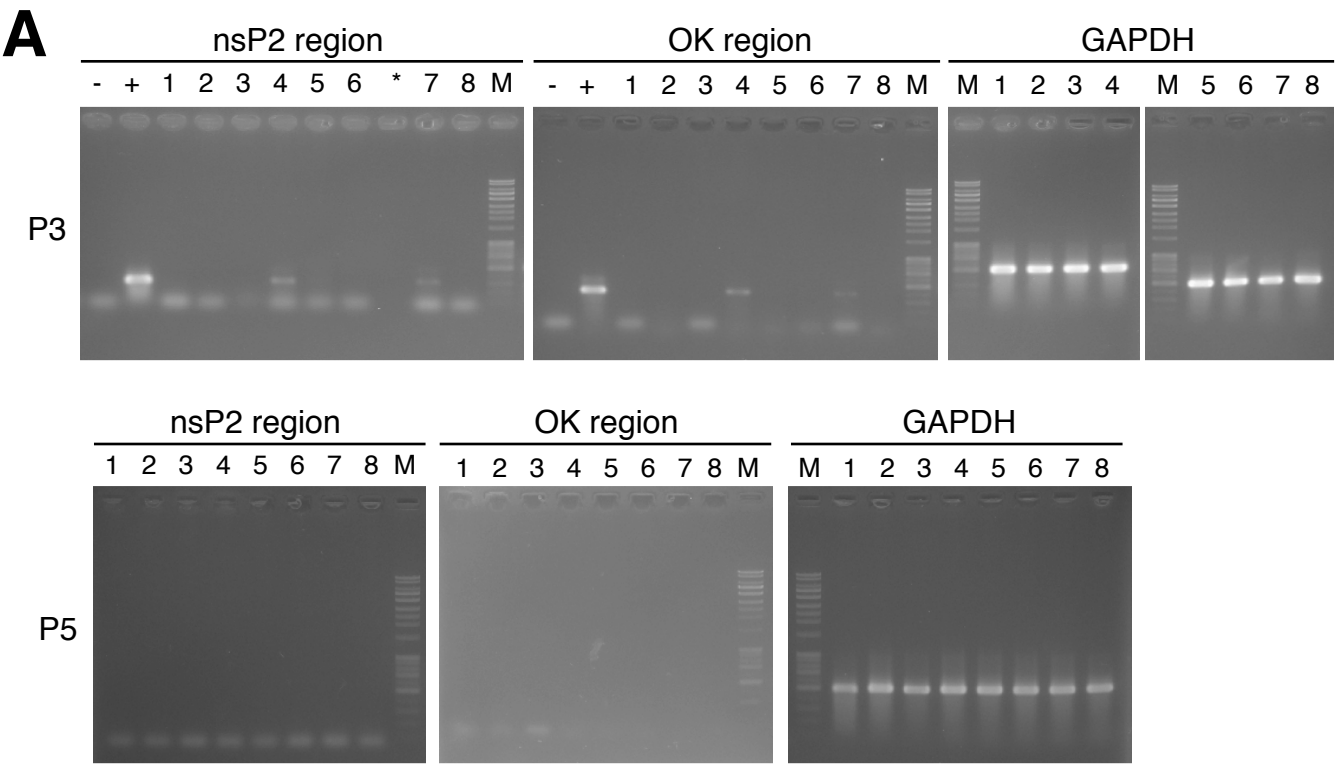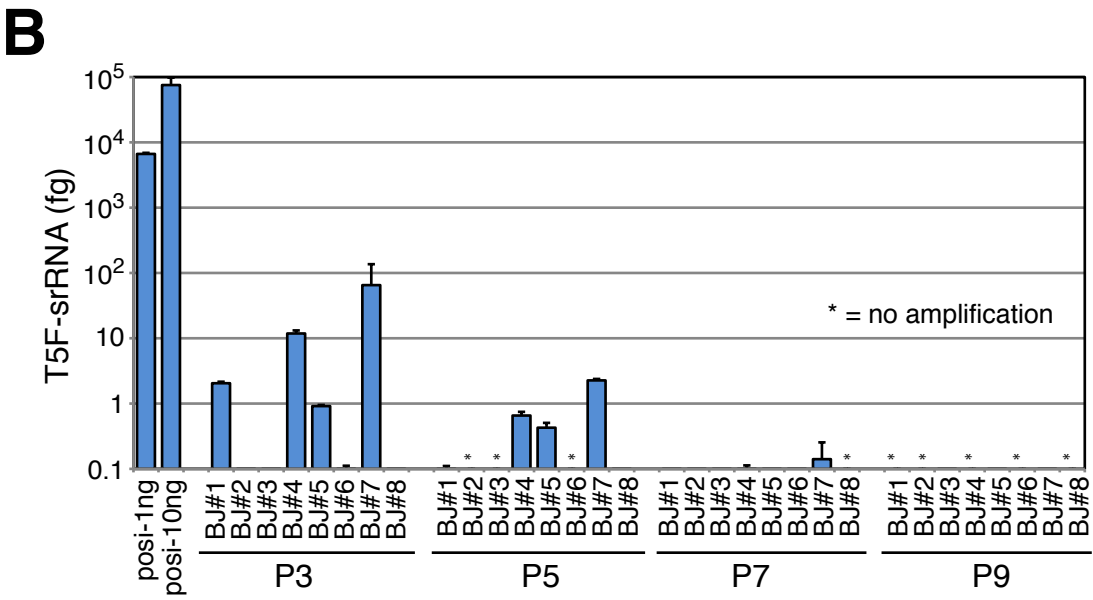

Supplement: S2 Fig — A qRT-PCR analysis of 5F-srRNA in BJ-5F-srRNA iPSC clones. "-" = BJ cells without sr-RNA (negative control); "+" = srRNA transfected BJ cells (positive control). BJ-5F-clones (1–8). iPSC clones were mechanically isolated and cultured in the absence of B18R. Total RNA was isolated from Passage 3 and 5, and used for qRT-PCR. Primers were in nsP2 region and Oct4-Klf4 (OK) region. B qRT-PCR analysis with TaqMan probe for srRNA (nsP1 region) was performed in BJ-5F-srRNA clones (1–8). * = no amplification. (PDF) [file pone.0182018.s002.pdf]

**A**

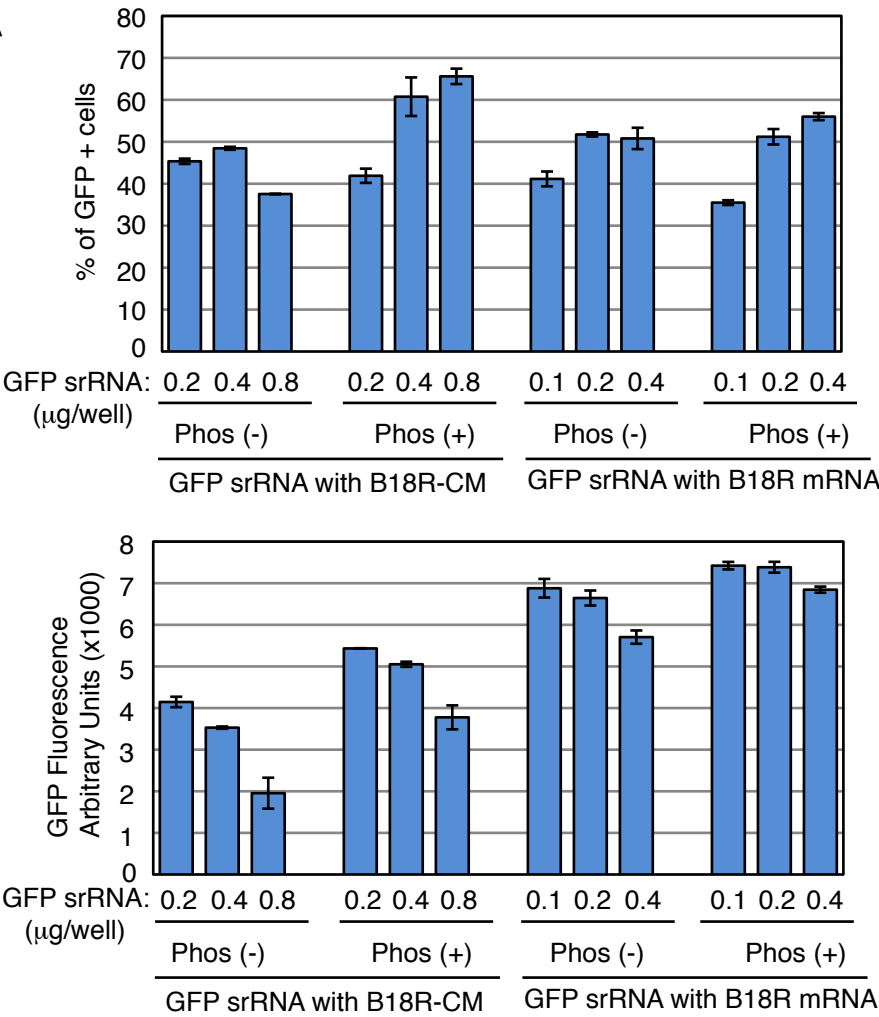

**C**

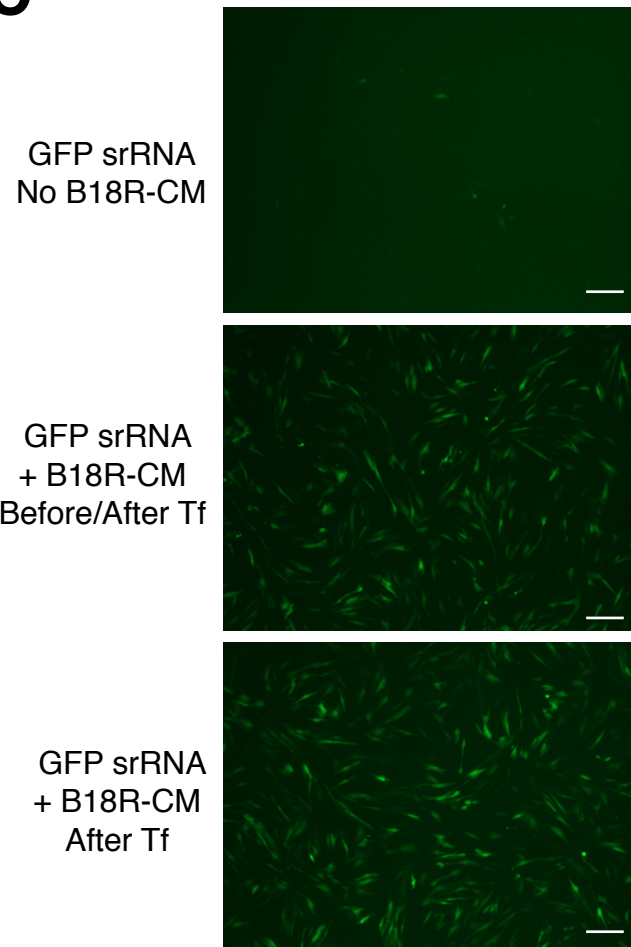

**B**

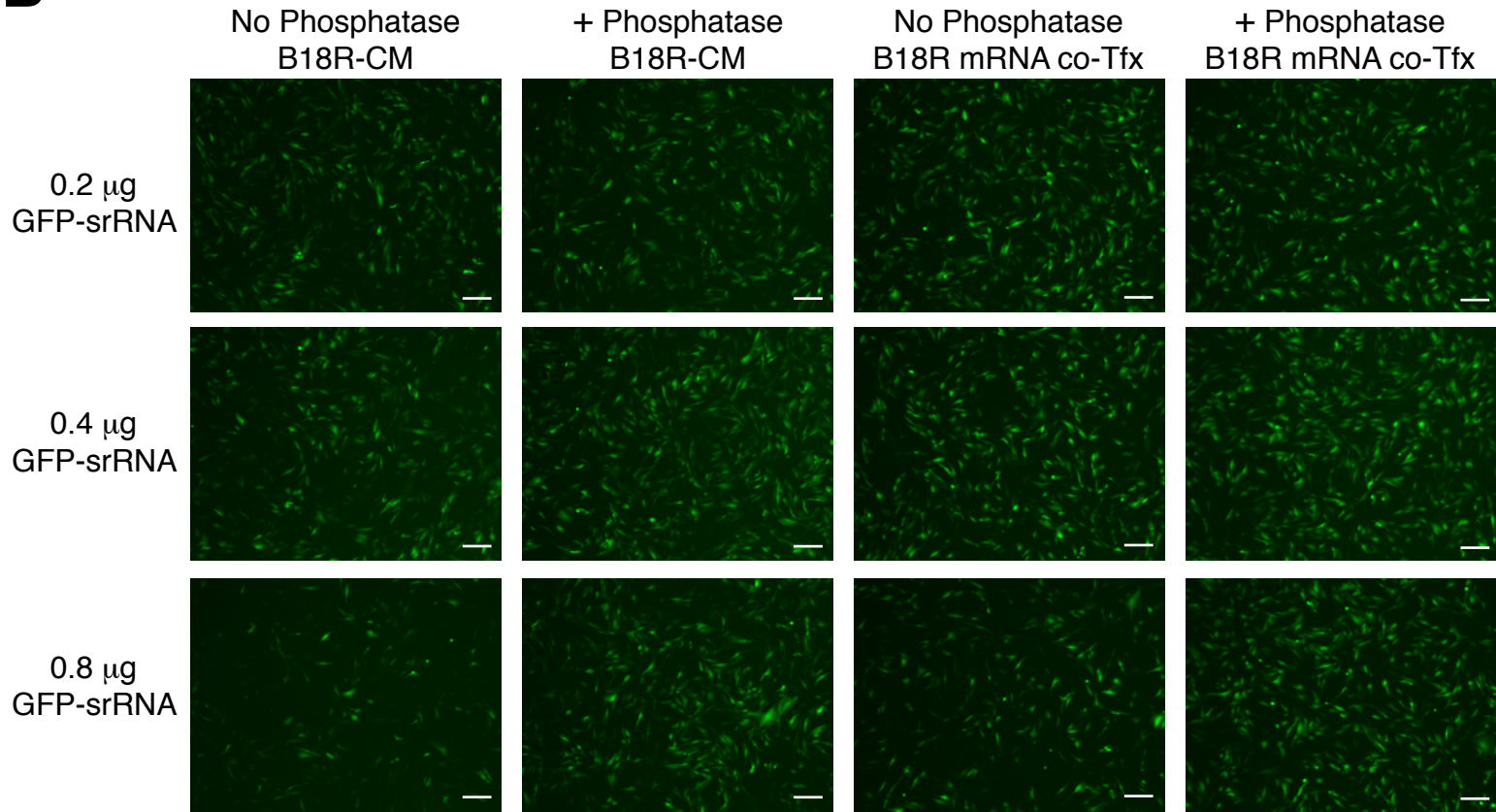

Supplement: S3 Fig — A OCT4 expression with phosphatase treated 5F-sRNA. 5F-srRNA was prepared with or without phosphatase treatment. 5F-srRNA and B18R mRNA were co-transfected into BJ cells with Lipofectamine 2000 for Western blotting. 5F-Phos (-): no phosphatase treated 5F-srRNA; 5F-Phos(+): phosphatase treated 5F-srRNA. B TagGFP2 srRNA (gifted from EMD Millipore) was prepared with or without phosphatase treatment. TagGFP2 srRNA was transfected into BJ cells with MessengerMax reagent in the presence of recombinant B18R protein. One day after the transfection, GFP expression was measured by FACS. Phos (+): Phosphatase treated srRNA; Phos (-): No phosphatase treated srRNA. C Microphotographs of cells from (A). Scale bar, 250 μm. (PDF) [file pone.0182018.s003.pdf]
