# Supplementary material for: Enhanced generation of iPSCs from older adult human cells by a synthetic five-factor self-replicative RNA
Source: PLoS One. 2017 Jul 27;12(7):e0182018. doi: 10.1371/journal.pone.0182018 (PMC5531586; doi:10.1371/journal.pone.0182018)
Supplement: S1 Table — (PDF) [file pone.0182018.s004.pdf]

Supporting Information Table S1. Oligonucleotides Used for Plasmid Constructs.

Oligo name	Oligonucleotide Sequence	
P2A Forward	5’-AATTCCAATTGACCGGTTCCGGAGCCACGAAC TTCTCTCTGTTAAAGCAAGCAGGAGACGTGGAAG AAAACCCCGGTCCTG	For P2A peptide plasmid
P2A Reverse	5’-GATCCAGGACCGGGGTTTTCTTCCACGTCTCCTG CTTGCTTTAACAGAGAGAAGTTCGTGGCTCCGGAAC CGGTCAATTGG	For P2A peptide plasmid
EcoR1-Mfe1-cMyc	5’-ggaattccaattggccaccATGCCCCTCAACGTTAGCTTC	For iML plasmid
BamH1-Age1-cMyc	5’-cgggatccaccggtCGCACAAGAGTTCCGTAGCTG	For iML plasmid
EcoR1-Mfe1-Glis1	5’-ggaattccaattggccaccATGGCAGAGGCCCGCACATCC	For iGL, iGM plasmids
BamH1-Age1-Glis1	5’-cgggatccaccggtGGTGTCTGTGTAGATGGAGG	For iGL, iGM plasmids
BamH1-Lin28A	5’-cgggatccATGGGCTCCGTGTCCAACCAGCAG	For iML, iGL plasmids
EcoR1-Not1-Stop-Lin28A	5’-ggaattcgcggccgcTCAATTCTGTGCCTCCGGGAGC	For iML, iGL plasmids
Xba1-BamH1-cMyc	5’-gctctagaggatccATGCCCCTCAACGTTAGCTTCACC	For iGM plasmid
EcoR1-Not1-Stop-cMyc	5’-ggaattcgcggccgcTCACGCACAAGAGTTCCGTAGCTG	For iGM plasmid
