# Supplementary material for: Enhanced generation of iPSCs from older adult human cells by a synthetic five-factor self-replicative RNA
Source: PLoS One. 2017 Jul 27;12(7):e0182018. doi: 10.1371/journal.pone.0182018 (PMC5531586; doi:10.1371/journal.pone.0182018)
Supplement: S2 Table — (PDF) [file pone.0182018.s005.pdf]

**Supporting information Table S2. srRNA Generation of iPS Cells from Neonatal Human Fibroblasts**

| | srRNA | Cell | Transfection Condition | Other Condition | Day of Passage | Number of AP+ Colonies | Day of AP Staining |
|---|---|---|---|---|---|---|---|
| Exp. 1 | 4F OKS-iM | BJ | 1 μg srRNA | - | Day 10 | 9 | Day 21 |
| Exp. 1 | 4F OKS-iM | BJ | 2 μg srRNA | - | Day 10 | 16 | Day 21 |
| Exp. 1 | 4F OKS-iG | BJ | 1 μg srRNA | - | Day 10 | 14 | Day 21 |
| Exp. 1 | 4F OKS-iG | BJ | 2 μg srRNA | - | Day 10 | 26 | Day 21 |
| Exp. 1 | 5F OKS-iGM | BJ | 1 μg srRNA | - | Day 10 | 96 | Day 21 |
| Exp. 1 | 5F OKS-iGM | BJ | 2 μg srRNA | - | Day 10 | 81 | Day 21 |
| Exp. 1 | 6F OKS-iGML | BJ | 1 μg srRNA | - | Day 10 | 40 | Day 21 |
| Exp. 1 | 6F OKS-iGML | BJ | 2 μg srRNA | - | Day 10 | 23 | Day 21 |
| Exp. 1 | 4F OKS-iM | BJ | 1 μg srRNA | - | Day 10 | 11 | Day 28 |
| Exp. 1 | 4F OKS-iM | BJ | 2 μg srRNA | - | Day 10 | 19 | Day 28 |
| Exp. 1 | 4F OKS-iG | BJ | 1 μg srRNA | - | Day 10 | 29 | Day 28 |
| Exp. 1 | 4F OKS-iG | BJ | 2 μg srRNA | - | Day 10 | 76 | Day 28 |
| Exp. 1 | 5F OKS-iGM | BJ | 1 μg srRNA | - | Day 10 | 183 | Day 28 |
| Exp. 1 | 5F OKS-iGM | BJ | 2 μg srRNA | - | Day 10 | 137 | Day 28 |
| Exp. 1 | 6F OKS-iGML | BJ | 1 μg srRNA | - | Day 10 | 104 | Day 28 |
| Exp. 1 | 6F OKS-iGML | BJ | 2 μg srRNA | - | Day 10 | 64 | Day 28 |
| | | | | | | | |
| Exp. 2 | 4F OKS-iM | BJ | 1 μg srRNA | - | Day 10 | 13 | Day 23 |
| Exp. 2 | 4F OKS-iM | BJ | 1 μg srRNA | - | Day 10 | 41 | Day 23 |
| Exp. 2 | 4F OKS-iG | BJ | 1 μg srRNA | - | Day 10 | 69 | Day 23 |
| Exp. 2 | 4F OKS-iG | BJ | 1 μg srRNA | - | Day 10 | 72 | Day 23 |
| Exp. 2 | 5F OKS-iGM | BJ | 1 μg srRNA | - | Day 10 | 310 | Day 23 |
| Exp. 2 | 5F OKS-iGM | BJ | 1 μg srRNA | - | Day 10 | 290 | Day 23 |
| Exp. 2 | 4F OKS-iM | BJ | 1 μg srRNA | Passage 18 | Day 10 | 5 | Day 23 |
| Exp. 2 | 4F OKS-iG | BJ | 1 μg srRNA | Passage 18 | Day 10 | 7 | Day 23 |
| Exp. 2 | 5F OKS-iGM | BJ | 1 μg srRNA | Passage 18 | Day 10 | 63 | Day 23 |
| Exp. 2 | 4F OKS-iM | HFF | 1 μg srRNA | Passage 25 | Day 10 | 8 | Day 23 |
| Exp. 2 | 4F OKS-iG | HFF | 1 μg srRNA | Passage 25 | Day 10 | 15 | Day 23 |
| Exp. 2 | 5F OKS-iGM | HFF | 1 μg srRNA | Passage 25 | Day 10 | 148 | Day 23 |
| | | | | | | | |
| Exp. 3 | 5F OKS-iGM | BJ | 1 μg srRNA | - | Day 10 | 90 | Day 21 |
| Exp. 3 | 5F OKS-iGM | BJ | 2 μg srRNA | - | Day 10 | 82 | Day 21 |
| Exp. 3 | 5F OKS-iGM | BJ | 1 μg srRNA | Matrigel | Day 10 | 262 | Day 21 |
| Exp. 3 | 5F OKS-iGM | BJ | 2 μg srRNA | Matrigel | Day 10 | 167 | Day 21 |
| Exp. 3 | 5F OKS-iGM | BJ | 1 μg srRNA | Laminin | Day 10 | 350 | Day 21 |
| Exp. 3 | 5F OKS-iGM | BJ | 2 μg srRNA | Laminin | Day 10 | 206 | Day 21 |
| | | | | | | | |
| Exp. 4 | 5F OKS-iGM | BJ | 1 μg srRNA | - | Day 10 | 108 | Day 21 |
| Exp. 4 | 5F OKS-iGM | BJ | 1 μg srRNA | - | Day 10 | 178 | Day 28 |
| Exp. 4 | 5F OKS-iGM | BJ | 1 μg srRNA | Zeb, 60 μM | Day 10 | 251 | Day 21 |
| Exp. 4 | 5F OKS-iGM | BJ | 1 μg srRNA | Zeb, 60 μM | Day 10 | 313 | Day 28 |
| Exp. 4 | 5F OKS-iGM | BJ | 1 μg srRNA | Zeb, 100 μM | Day 10 | 162 | Day 21 |
| Exp. 4 | 5F OKS-iGM | BJ | 1 μg srRNA | Zeb, 100 μM | Day 10 | 223 | Day 28 |

Cells were passaged on 6-well plate on day 0 and cultured to 80-100% confluency on day 1. To minimize the IFN responses, cells were treated with 20 % B18R-CM 20 min before the transfection. The srRNA was transfected with Lipofectamine 2000 in the presence of 20% B18R-CM (No B18R mRNA co-transfection). Medium was changed to the Advanced DMEM containing 20% B18R-CM after 3hr transfection. ES culture medium was used from day 7. Cells were passaged on feeder cells as indicated day except for Exp.2 (feeder free condition). Zebulaline (Zeb) was treated from day 2 to day 10. Passage 8 to 10 of BJ cells was used. Colonies were stained with Alkaline Phosphatase (AP) and the numbers of AP positive colonies per starting well were indicated.
