# Supplementary material for: Enhanced generation of iPSCs from older adult human cells by a synthetic five-factor self-replicative RNA
Source: PLoS One. 2017 Jul 27;12(7):e0182018. doi: 10.1371/journal.pone.0182018 (PMC5531586; doi:10.1371/journal.pone.0182018)
Supplement: S3 Table — (PDF) [file pone.0182018.s006.pdf]

Supporting Information Table S3. 5F-srRNA Generation of iPSCs by MessengerMAX Transfection in BJ Cells

Transfection Reagent		Puro selection	Day of Passage	Passage plate	Number of AP+ Colonies
mMAX	2 μg srRNA	+	Day 8	feeder	789
mMAX	4 μg srRNA	+	Day 8	feeder	615
L2K	2 μg srRNA	+	Day 8	feeder	364
L2K	4 μg srRNA	+	Day 8	feeder	258
mMAX	2 μg srRNA	-	Day 5	feeder	306
mMAX	4 μg srRNA	-	Day 5	feeder	291
L2K	2 μg srRNA	-	Day 5	feeder	101
L2K	4 μg srRNA	-	Day 5	feeder	104
mMAX	2 μg srRNA	+	Day 8	feeder	759
mMAX	4 μg srRNA	+	Day 8	feeder	660
mMAX	2 μg srRNA	+	Day 8	Matrigel	642
mMAX	4 μg srRNA	+	Day 8	Matrigel	486
mMAX	2 μg srRNA	+	Day 8	Laminin	720
mMAX	4 μg srRNA	+	Day 8	Matrigel	567
mMAX	2 μg srRNA	-	Day 5	feeder	318
mMAX	4 μg srRNA	-	Day 5	feeder	327
mMAX	2 μg srRNA	-	Day 5	Matrigel	117
mMAX	4 μg srRNA	-	Day 5	Matrigel	153
mMAX	2 μg srRNA	-	Day 5	Laminin	102
mMAX	4 μg srRNA	-	Day 5	Matrigel	108

BJ cells (Passage 8) were plated on 6-well on day 0 and cultured to 100% confluency on day 1. Cells were co-transfected with 5F srRNA plus B18R mRNA (1:1 ratio, 2 or 4 μg RNA/well) in the absence of serum and B18R-CM. After 3 hr, medium was changed to the Advanced DMEM containing 20% B18R-CM. ES culture medium was used starting on day 5. Cells were passaged onto feeder cells or Matrigel as indicated. Colonies were stained with Alkaline Phosphatase (AP) on day 24 and numbers of AP positive colonies per starting well are reported. mMAX = MessengerMAX transfection reagent; L2K = Lipofectamine 2000 transfection reagent.
